# Exploring machine learning algorithms for predicting fertility preferences among reproductive age women in Nigeria

**DOI:** 10.3389/fdgth.2024.1495382

**Published:** 2025-01-16

**Authors:** Zinabu Bekele Tadese, Teshome Demis Nimani, Kusse Urmale Mare, Fetlework Gubena, Ismail Garba Wali, Jamilu Sani

**Affiliations:** ^1^ Department of Health Informatics, College of Medicine and Health Science, Samara University, Samara, Ethiopia; ^2^ Department of Epidemiology and Biostatistics, School of Public Health College of Medicine and Health Science, Haramaya University, Harar, Ethiopia; ^3^ Department of Nursing, College of Medicine and Health Sciences, Samara University, Samara, Ethiopia; ^4^ Department of Public Health, College of Medicine and Health Science, University of Gondar, Gondar, Ethiopia; ^5^ Department of Demography & Social Statistics, Federal University, Birnin-Kebbi, Kebbi State, Nigeria

**Keywords:** fertility preference, Demographic and Health Survey, Nigeria, machine learning (ML), maternity

## Abstract

**Background:**

Fertility preferences refer to the number of children an individual would like to have, regardless of any obstacles that may stand in the way of fulfilling their aspirations. Despite the creation and application of numerous interventions, the overall fertility rate in West African nations, particularly Nigeria, is still high at 5.3% according to 2018 Nigeria Demographic and Health Survey data. Hence, this study aimed to predict the fertility preferences of reproductive age women in Nigeria using state-of-the-art machine learning techniques.

**Methods:**

Secondary data analysis from the recent 2018 Nigeria Demographic and Health Survey dataset was employed using feature selection to identify predictors to build machine learning models. Data was thoroughly assessed for missingness and weighted to draw valid inferences. Six machine learning algorithms, namely, Logistic Regression, Support Vector Machine, K-Nearest Neighbors, Decision Tree, Random Forest, and eXtreme Gradient Boosting, were employed on a total sample size of 37,581 in Python 3.9 version. Model performance was assessed using accuracy, precision, recall, F1-score, and area under the receiver operating characteristic curve (AUROC). Permutation and Gini techniques were used to identify the feature's importance.

**Results:**

Random Forest achieved the highest performance with an accuracy of 92%, precision of 94%, recall of 91%, F1-score of 92%, and AUROC of 92%. Factors influencing fertility preferences were number of children, age group, and ideal family size. Region, contraception intention, ethnicity, and spousal occupation had a moderate influence. The woman's occupation, education, and marital status had a lower impact.

**Conclusion:**

This study highlights the potential of machine learning for analyzing complex demographic data, revealing hidden factors associated with fertility preferences among Nigerian women. In conclusion, these findings can inform more effective family planning interventions, promoting sustainable development across Nigeria.

## Background

Fertility preferences, or desire, refer to the number of children that an individual would like to have, regardless of any obstacles that may stand in the way of fulfilling their aspirations (1). These preferences are dynamic rather than constant, often changing due to life circumstances, policy interventions, or broader societal trends (2). Studies on the stability of fertility preferences have critiqued the measurement of fertility preferences, demonstrating that fertility preferences do not remain constant throughout life (3). Over the past 50 years, one of the most significant developments in the demography of low- and middle-income countries (LMICs) has been the rising prevalence of modern contraception (4). Changes in the use of contraceptives have been intensely debated in both scientific and policy settings because they directly address the policy question of expanding family planning services (5). During the COVID-19 pandemic, the fertility preferences of women started to change, leading to a decrease in national fertility rates (6). This shift has been attributed to the financial and emotional burdens of parenting, the influence of their partners' desires, and the stability of their relationships (7).

In sub-Saharan Africa (SSA), most nations have a fertility rate of more than five children per woman (8). While the total number of births per woman in SSA in 2022 was 4.5, estimates for the Arab world, South Asia, Latin America and the Caribbean, and the European Union were 3.1, 2.2, 1.8, and 1.5, respectively (9). High fertility rates contribute to increased maternal and child health burdens, strain public health infrastructure, and slow progress toward achieving global development goals, such as those outlined in the Sustainable Development Goals (SDGs) (10–12).

Despite the development and implementation of multiple treatments, the general fertility rate in West African countries remains high. Efforts to reduce fertility rates in Nigeria include the National Population Policy, which advocates voluntary fertility regulation to achieve economic and social development goals (13). As a result, according to the Nigeria Demographic and Health Survey (NDHS), the fertility rate declined from 6.0 in 1990 to 5.3 in 2018 (14). Regardless of such initiatives, many factors, including limited access to contraception, low levels of female education, and deeply ingrained cultural norms, continue to sustain high fertility preferences. Understanding these preferences and the factors that influence them is critical for designing effective interventions. Previous research has predominantly relied on traditional statistical methods, such as logistic regression, to analyze predictors of fertility preferences (2, 13, 15, 16). While these methods are valuable for hypothesis-driven analyses, they are often constrained by their inability to model complex, non-linear relationships between predictors and outcomes. For example, demographic and health variables such as age, marital status, education, and contraceptive use may interact in ways that are not easily captured by linear models (10, 17, 18). In addition, traditional methods often require strong assumptions about the data distribution, which may not hold in real-world demographic datasets (19, 20). Machine learning (ML) has emerged as a transformative tool in demographic research, offering advanced techniques to identify hidden patterns within large datasets (21, 22). This study addresses this gap by employing ML algorithms to predict fertility preferences using nationally representative data from the 2018 NDHS. Furthermore, this study contributes to the growing body of literature on ML in demographic research by providing a novel framework for predicting fertility preferences.

## Methods

### Study setting and design

The NDHS employed a population-based cross-sectional study design to collect the data in two stages using a stratified sampling technique to select the study participants. This study used a predictive modeling approach to predict the fertility preference of reproductive age women based on data obtained from the NDHS.

### Study population

All women aged 15–49 years from the 2018 NDHS were the study population.

### Data source and sample size

This study utilizes data from the 2018 NDHS, obtained with authorization through an online request system on their official website (https://dhsprogram.com/). The study focuses on the individual record (IR) file, extracting both dependent and independent variables relevant to fertility preferences. An actual sample size of 37,581 was considered for this analysis of women who expressed their desire to either “have another” child or “no more” children.

### Study variables

This study defines the outcome variable as “fertility preference” among Nigerian women. This variable is encoded in binary, with “0” indicating a desire for “no more children” and “1” indicating a desire to “have another child.” The original NDHS data categorized this variable into five options. However, the analysis excluded the remaining categories—“undecided,” “sterilized,” and “declared infecund”—as they do not give unambiguous information on current fertility preference.

The explanatory variables in the study include demographic, socioeconomic, and health factors. They are age, region, woman's education level, ethnicity, wealth index, number of children ever born, birth intentions in the next 5 years, contraceptive use intention, marital status, age at first sexual intercourse, ideal number of children, husband's occupation, and woman’s occupation.

### Data management and analysis

#### Data pre-processing

The raw NDHS data underwent extensive pre-processing to ensure its suitability for machine learning analysis. ML algorithms perform best with clean, complete, and balanced datasets where the number of observations in each category is comparable (23).

The initial analysis revealed a class imbalance in the target variable (fertility preference). Specifically, the class representing women who desired “no more children” (coded as 0) was significantly under-represented compared to those wanting “another child” (coded as 1). To address this, the synthetic minority oversampling technique (SMOTE) was employed. SMOTE creates synthetic data points for the minority class (“no more children”), effectively balancing the class distribution and improving the model's ability to learn from both preferences.

Missing data less than 10% were handled by multiple imputations by multivariate imputation by chained equations (MICE) after checking the type of missing type [missing completely at random (MCAR), missing at random (MAR), or missing not at random (MNAR)]. Continuous variables were transformed into categorical bins, and variables with low-frequency categories were recategorized. These pre-processing steps ensured minimal missingness, near-complete data for all included variables, and improved compatibility with the chosen machine learning algorithms, ultimately leading to a robust and reliable analysis of fertility preferences among Nigerian women.

To identify the most informative variables influencing fertility preferences among Nigerian women, this study employed a multi-step feature selection process. The process began by leveraging existing knowledge and exploratory data analysis (EDA) techniques such as descriptive statistics and visualization to assess data distribution and relationships between variables and outliers. Bivariate logistic regression was then used to analyze the association between each feature and the outcome variable. This method provided a more comprehensive understanding of how each feature impacts the probability of a woman wanting “no more children” compared to wanting “another child.” Following this initial screening, recursive feature elimination (RFE) was implemented, which is a machine learning technique that iteratively removes the least informative features. In addition, a correlation heatmap was then used to identify and eliminate highly correlated features within the continuous feature set to avoid multicollinearity and potential model instability. By incorporating these rigorous steps alongside domain knowledge, this methodology ensured the selection of the most relevant and informative features for building the final prediction model for fertility preferences.

Following the feature selection process, we employed two key techniques to evaluate the relative importance of the chosen features within the final model: permutation importance and Gini importance. Permutation importance is a reliable method that stands out for its model-agnostic nature. It works by randomly shuffling the values of each feature and observing the resulting decrease in model performance (24). Features that lead to a substantial decline in performance when shuffled are deemed more critical for the model's predictions. This method offers a broad understanding of feature importance across different models. In contrast, Gini importance is particularly valuable for understanding feature significance within decision tree models, such as the random forest model used in this study. It quantifies the frequency with which a feature is used to partition the data at each decision node within the individual trees of the random forest. Features that consistently appear at these decision points are considered more influential in the model's final prediction of fertility preference (25).

#### Model development and evaluation

This study implemented six machine learning algorithms in Python 3.9 to develop a model for predicting fertility preference. The selection of six ML algorithms, which include Logistic Regression (LR), Support Vector Machine (SVM), K-Nearest Neighbors (KNN), Decision Tree (DT), Random Forest (RF), and eXtreme Gradient Boosting (XGBoost), was intentional to capture diverse modeling paradigms (26, 27). Logistic Regression serves as a baseline linear model, while SVM is well-suited for capturing non-linear decision boundaries. KNN, an instance-based method, leverages distance metrics to classify observations, while Decision Tree offers high interpretability. Ensemble methods such as Random Forest and XGBoost, known for their robustness and ability to handle structured datasets, are included to enhance predictive accuracy (26, 28). To ensure the model generalizes well to unseen data, which is a crucial step in the machine learning process, the pre-processed data was split into training (80%) and testing (20%) sets. The training data were used to fit the model, where the target variable (fertility preference) was what the model was predicting, and the selected features (factors influencing fertility preference) acted as predictors. The model's performance was then evaluated on the unseen testing data using various metrics [accuracy, precision, recall, F1-score, and area under the receiver operating characteristic curve (AUROC)] and confusion matrix results. In addition, stratified k-fold cross-validation was employed to further assess the model's generalizability by training and evaluating it on multiple random splits of the data. This evaluation of unseen data helps prevent overfitting, where the model performs well on the training data but fails to generalize to new data. To identify the best model for predicting fertility preference, a comprehensive evaluation using various performance metrics such as accuracy, precision, sensitivity (recall), and F1-score was conducted. These metrics assess the performance of each model in accurately classifying cases based on their fertility preference. To achieve this, a confusion matrix was used to identify true positives (TPs), false positives (FPs), true negatives (TNs), and false negatives (FNs) and all performance metrics were calculated based on these values.

#### Generalizability assessment using cross-validation

**Figure 1:**
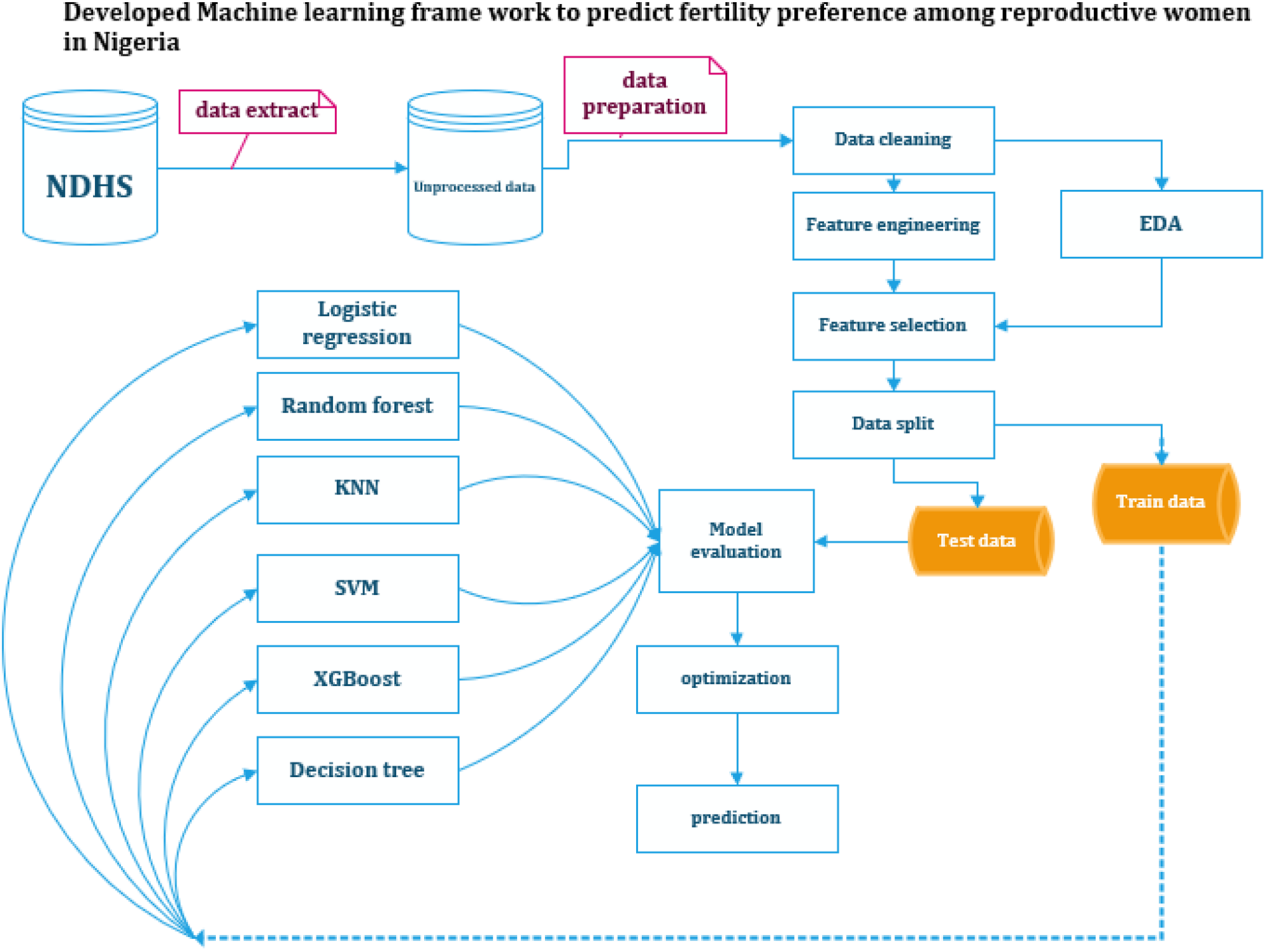
Summary of the applied framework for predicting fertility preference among reproductive age women in Nigeria. NDHS, Nigeria Demographic and Health Survey; EDA, exploratory data analysis; SVM, Support Vector Machine; KNN, K-Nearest Neighbors; XGBoost, eXtreme Gradient Boosting. Created using Microsoft Visio.

In addition to the metrics above, stratified k-fold cross-validation was employed to assess the generalizability of the models. This technique involves splitting the data into multiple folds while ensuring each fold preserves the original data's class distribution (desire for another child). Training and evaluating the model on these stratified folds provides a more robust estimate of its performance on unseen data, especially for imbalanced datasets. This evaluation of unseen data helps prevent overfitting, where the model performs well on the training data but fails to generalize to new data (Figure 1).

### Statistical analysis

A comprehensive statistical analysis using STATA 15 was conducted as a crucial preliminary step to understand fertility preference and enhance the subsequent machine learning models. This analysis served three key purposes. First, exploring the distribution of fertility preference across key variables with descriptive statistics and cross-tabulations provided insights into factors influencing fertility preference. Second, the statistical analysis informed the selection of features for the machine learning models. Finally, logistic regression analysis was employed to explore further the relationship between key variables and the outcome variable (fertility preference). This analysis, particularly focusing on adjusted odds ratios (aORs), identified statistically significant associations between several variables and the desire for another child. By integrating these statistical methods, the analysis not only enriched the understanding of fertility dynamics but also laid the groundwork for robust and reliable predictive machine learning models.

## Results

### Sociodemographic characteristics of respondents

Table 1 presents the sociodemographic characteristics of the 37,581 women aged 15–49 years included in the study. Women aged 15–29 years constituted 57.91% of the respondents. The participants were distributed across the six geopolitical zones of Nigeria, with the North West having the highest proportion (23.79%). Most respondents (59.19%) resided in rural areas. Regarding education, nearly two-thirds (64.1%) had at least a primary education. The wealth index distribution showed a relatively even spread across the five categories. Contraceptive use was prevalent among 13.63% of the respondents, with a significant proportion (34.83%) intending to use contraceptives in the future. The majority (66.21%) had exposure to media. The reproductive history of the respondents varied, with a substantial proportion (42.03%) having one or fewer children.

**Table 1:** Sociodemographic characteristics of reproductive age women in Nigeria in 2018 (*N* = 37,581).

Variable	Category	Weighted frequency	Percentage
Age (years)	15–19	7,552	20.10
	20–24	6,432	17.12
	25–29	6,724	17.89
	30–34	5,457	14.52
	35–39	4,765	12.68
	40–44	3,452	9.19
	45–49	3,199	8.51
Region	North Central	7,108	18.91
	North East	6,879	18.30
	North West	8,939	23.79
	South East	5,034	13.40
	South	4,476	11.91
	South West	5,145	13.69
Residence	Urban	15,335	40.81
	Rural	22,246	59.19
Educational level	No education	12,731	33.88
	Primary	5,728	15.24
	Secondary	15,147	40.30
	Higher	3,975	10.58
Wealth index	Poorest	6,869	18.28
	Poorer	7,478	19.90
	Middle	8,013	21.32
	Richer	7,960	21.18
	Richest	7,261	19.32
Contraceptive use	Using modern methods	3,855	10.26
	Using traditional methods	1,268	3.37
	Intends to use	13,088	34.83
	Intends not to use	19,370	51.54
Number of children	0–1	15,795	42.03
	2–3	9,353	24.89
	4–5	7,304	19.44
	6+	5,129	13.65
Births in 5 years	0	17,496	46.56
	1	10,440	27.78
	2	8,200	21.82
	3+	1,445	3.85
Media exposure	no	12,697	33.79
	yes	24,884	66.21

### Inferential statistics

Table 2 illustrates the inferential statistics analysis with logistic regression. This analysis reveals the strong relationships between sociodemographic factors and fertility desires.

**Table 2:** Sociodemographic characteristics and association with fertility preference.

Variable	Fertility preference		aOR (95% CI)	*p*-value
	No more freq. (%)	Want more freq. (%)		
Age (years)				
15–19	179 (2.1)	7,373 (25.5)	RC	
20–24	148 (1.7)	6,284 (21.8)	1.29 (0.85–1.97)	0.233
25–29	448 (5.2)	6,276 (21.7)	1.14 (0.77–1.69)	0.526
30–34	1,052 (12.1)	4,405 (15.3)	0.64 (0.43–0.94)	0.024
35–39	1,892 (21.8)	2,873 (9.9)	0.33 (0.22–0.49)	0.000
40–44	2,238 (25.8)	1,214 (4.2)	0.14 (0.09–0.20)	0.000
45–49	2,730 (31.4)	469 (1.6)	0.04 (0.03–0.06)	0.000
Region				
North Central	1,741 (20.0)	5,367 (18.6)	RC	
North East	1,072 (12.3)	5,807 (20.1)	2.87 (2.46–3.33)	0.000
North West	1,430 (16.5)	7,509 (26.0)	3.01 (2.59–3.49)	0.000
South East	1,584 (18.2)	3,450 (11.9)	0.91 (0.79–1.06)	0.220
South	1,261 (14.5)	3,215 (11.1)	0.86 (0.74–1.00)	0.051
South West	1,599 (18.4)	3,546 (12.3)	0.59 (0.51–0.68)	0.000
Educational level				
No education	2,894 (33.3)	9,837 (34.1)	RC	
Primary	2,203 (25.4)	3,525 (12.2)	0.80 (0.71–0.91)	0.001
Secondary	2,769 (31.9)	12,378 (42.8)	0.81 (0.71–0.93)	0.003
Higher	821 (9.5)	3,154 (10.9)	0.78 (0.64–0.95)	0.013
Wealth index				
Poorest	1,275 (14.7)	5,594 (19.4)	RC	
Poorer	1,624 (18.7)	5,854 (20.3)	0.83 (0.72–0.95)	0.007
Middle	1,985 (22.9)	6,028 (20.9)	0.77 (0.67–0.90)	0.001
Richer	2,023 (23.3)	5,937 (20.6)	0.76 (0.65–0.89)	0.001
Richest	1,780 (20.5)	5,481 (19.0)	0.70 (0.59–0.84)	0.000
Contraceptive use				
Modern methods	1,486 (17.1)	2,369 (8.2)	RC	
Traditional methods	504 (5.8)	764 (2.6)	1.12 (0.92–1.37)	0.259
Intends to use	1,787 (20.6)	11,301 (39.1)	1.87 (1.65–2.13)	0.000
Intends not to use	4,910 (56.5)	14,460 (50.0)	2.19 (1.93–2.47)	0.000
Number of children				
0–1	432 (5.0)	15,363 (53.2)	RC	
2–3	1,572 (18.1)	7,781 (26.9)	0.10 (0.08–0.13)	0.000
4–5	3,366 (38.8)	3,938 (13.6)	0.02 (0.02–0.03)	0.000
6+	3,317 (38.2)	1,812 (6.3)	0.01 (0.01–0.01)	0.000
Births in 5 years				
0	4,563 (52.5)	12,933 (44.8)	RC	
1	2,391 (27.5)	8,049 (27.9)	1.82 (1.63–2.03)	0.000
2	1,450 (16.7)	6,750 (23.4)	2.37 (2.09–2.70)	0.000
3+	283 (3.3)	1,162 (4.0)	2.00 (1.62–2.46)	0.000

RC, reference category; aOR, adjusted odds ratio.

Age shows a clear negative association, with women aged 30–34 years having a 64% lower desire for more children (aOR = 0.64, 95% CI: 0.43–0.94, *p* = 0.024) compared to the youngest group (15–19 years). This trend strengthens further, with the 45-to-49-year-old group exhibiting a 96% lower desire for more children (aOR = 0.04, 95% CI: 0.03–0.06, *p* < 0.001). Significant regional disparities existed, with the North West and North East regions exhibiting a distinctly higher desire for additional children compared to the national average. Women in the North West were three times more likely to desire more children (aOR = 3.01, 95% CI: 2.59–3.49, *p* < 0.001), while the North East also showed a similar increase (aOR = 2.87). In contrast, those in the South West region were the least likely to desire more children (aOR = 0.59).

Educational attainment also played a significant role, with individuals who were educated having statistically lower odds of wanting more children compared to those with no education (reference category). The odds ratios for primary, secondary, and higher education were 0.80 (95% CI 0.71–0.91, *p* = 0.001), 0.81 (95% CI 0.71–0.93, *p* = 0.003), and 0.78 (95% CI 0.64–0.95, *p* = 0.013), respectively, indicating a 20%–22% decrease in the desire for more children with each increase in the level of education. Wealth followed a similar trend, with wealthier individuals (richest and middle income) being less likely to desire more children (aOR = 0.70, 95% CI: 0.59–0.84, *p* < 0.001; aOR = 0.77, 95% CI: 0.67–0.90, *p* = 0.001) compared to the poorest group (reference category, RC).

Contraceptive use was another significant factor influencing fertility preferences, with those intending not to use contraception more likely to want more children (aOR = 2.19, 95% CI: 1.93–2.47, *p* < 0.001) compared to those intending to use contraceptives (aOR = 1.87, 95% CI: 1.65–2.13, *p* < 0.001). This points to a strong association between family planning intention and fertility desire.

As expected, the number of children a woman already had significantly reduced the desire for more children. Women with 3–5 children were 90% less likely to want more children than those with 0–1 child (RC), with an aOR of 0.10 (95% CI: 0.80–0.13, *p* < 0.001). Women with 6 or more children had an odds ratio of 0.01 (95% CI: 0.01–0.01, *p* < 0.001), indicating a 99% decrease in their desire for more children compared to those with 0–1 children.

Interestingly, recent childbirth experiences also influenced fertility preferences. Women who had two births in the last 5 years were more likely to want another child (aOR = 2.37, 95% CI: 2.09–2.70, *p* < 0.001) compared to those with no births (RC). Similarly, women with three or more births in the last 5 years had demonstrated a greater desire for additional children (aOR = 2.00, 95% CI: 1.62–2.46, *p* < 0.001).

### Machine learning analysis

**Figure 2:**
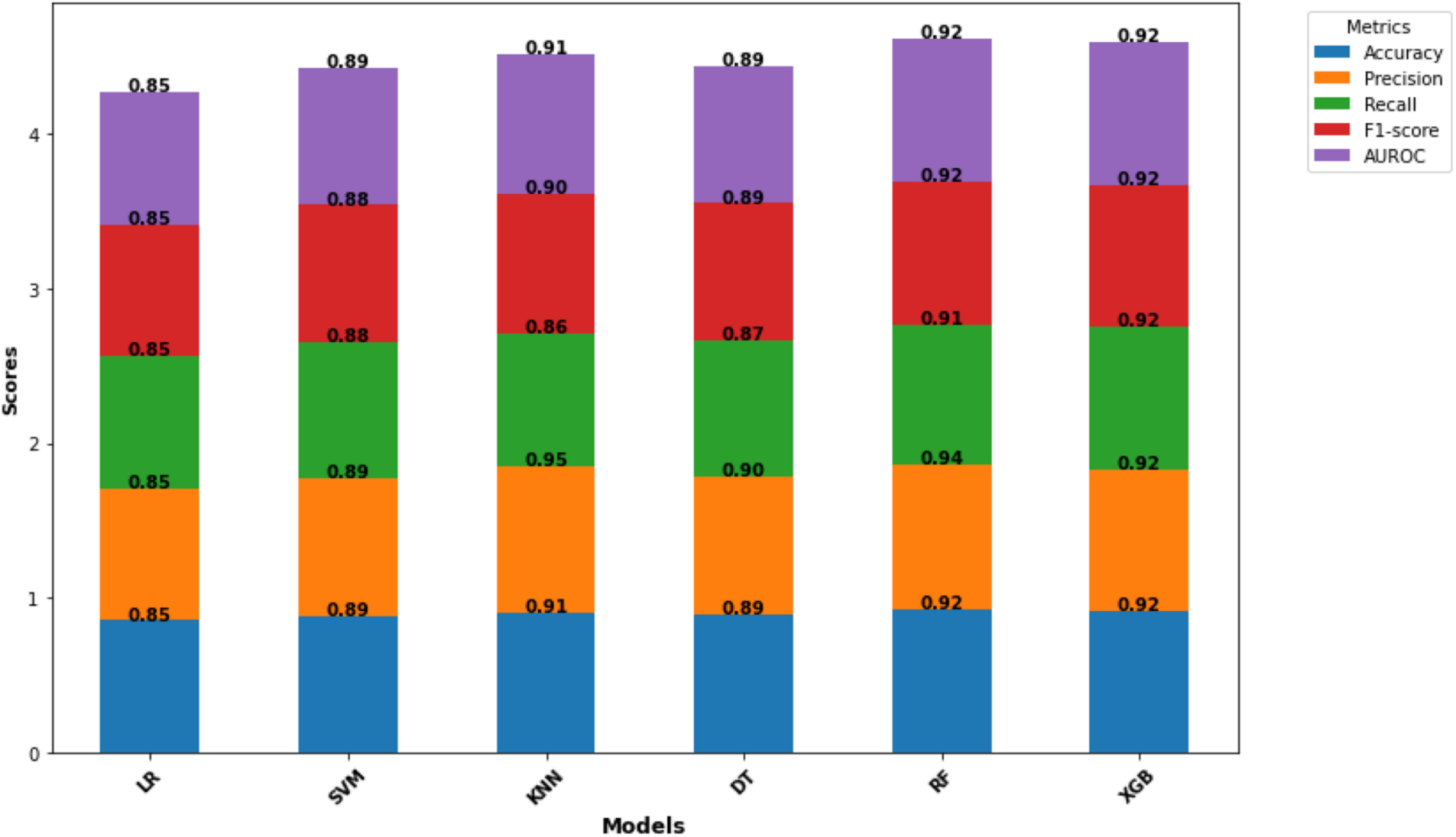
Model comparison and evaluation metrics. Copyright (c) 2024 Project Jupyter Contributors. All rights reserved. Licensed under BSD-3-Clause.

This study evaluated six machine learning models (LR, SVM, KNN, DT, RF, and XGBoost) to assess their ability to predict women's fertility preferences in Nigeria. The performance of each model was assessed using various metrics (accuracy, precision, recall, F1-score, and AUROC) and the confusion matrix results (Table 3). The machine learning evaluation analysis revealed exceptional performance from both Random Forest and XGBoost. These models achieved a high accuracy of 0.92 and well-balanced metrics across the board, solidifying their effectiveness in predicting fertility preferences. While both models excelled, the ROC curves revealed a slight advantage for Random Forest which has of area under the curve (AUC)-ROC of 0.98 compared to XGBoost's 0.97 (Figure 2). In addition, Random Forest exhibited a superior precision score (0.94) compared to XGBoost (0.92), indicating a stronger ability to accurately identify true positives (women who want more children) while minimizing false positives.

**Table 3:** Model comparison using confusion matrix and evaluation metrics.

	ML models					
	Logistic Regression	SVM	KNN	Decision Tree	Random Forest	XGBoost
Confusion matrix						
TP	4,989	5,196	5,559	5,260	5,475	5,338
FP	836	629	266	565	350	487
TN	4,876	5,038	4,906	5,013	5,198	5,265
FN	857	695	827	720	535	468
Evaluation metrics						
Accuracy	0.85	0.89	0.91	0.89	0.92	0.92
Precision	0.85	0.89	0.95	0.90	0.94	0.92
Recall	0.85	0.88	0.86	0.87	0.91	0.92
F1-score	0.85	0.88	0.90	0.89	0.92	0.92
AUROC	0.85	0.89	0.91	0.89	0.92	0.92

Given the research focus on minimizing false positives (mistakenly classifying women who do not desire more children as wanting more), Random Forest emerges as the preferred model. This prioritizes a cautious approach, reducing the risk of misclassification. While XGBoost demonstrated exceptional consistency with near-perfect recall and F1-score metrics, Random Forest's combined strengths in AUROC and precision, and its alignment with the research goal make it the optimal choice for this study.

**Figure 3:**
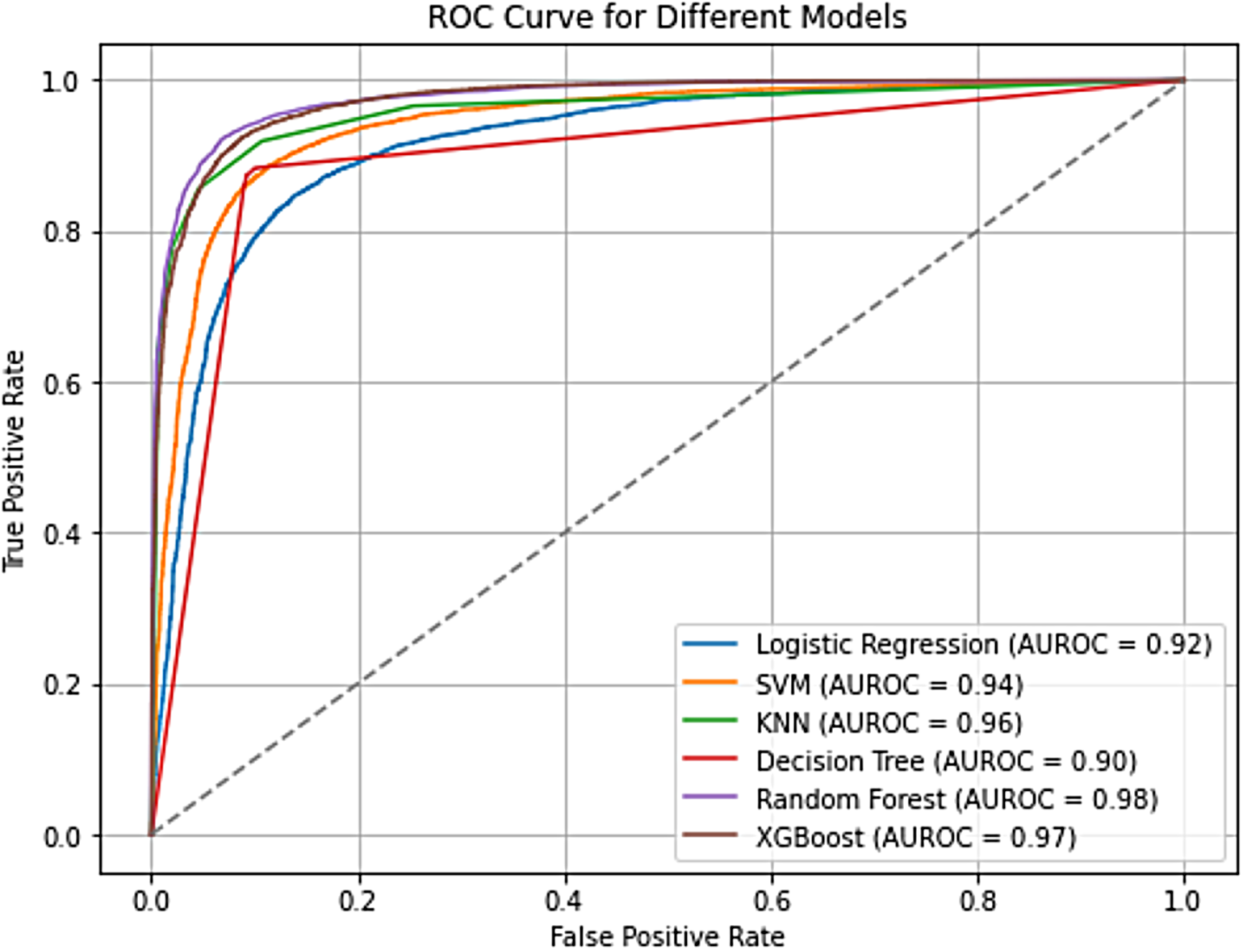
ROC curve for the predictive models. Copyright (c) 2024 Project Jupyter Contributors. All rights reserved. Licensed under BSD-3-Clause.

The performance of the six ML models employed for the study was further analyzed using ROC curves. The ROC curve is a fundamental tool in machine learning classification tasks, providing a visual representation of the model's ability to discriminate between positive and negative cases across various classification thresholds (24). It plots the true positive rate (TPR) of correctly identifying women desiring more children against the false positive rate (FPR) of incorrectly classifying women who do not. By analyzing the ROC curve and the AUROC, a valuable insight is gained into how effectively the model balances these competing factors, i.e., accurately identifying true positives while minimizing false positives. The ROC curve analysis reveals impressive performance across all six models in predicting the outcome, with AUROC values ranging from 0.90 to 0.98 (Figure 3). This signifies a strong overall ability to distinguish between women who want more children and those who do not. Among the models, Random Forest stands out with the highest AUROC score of 0.98, followed closely by XGBoost at 0.97 and KNN at 0.96.

### Features importance analysis

This analysis utilizes two feature importance techniques to shed light on these factors within the Random Forest model.

#### Permutation feature importance

**Figure 4:**
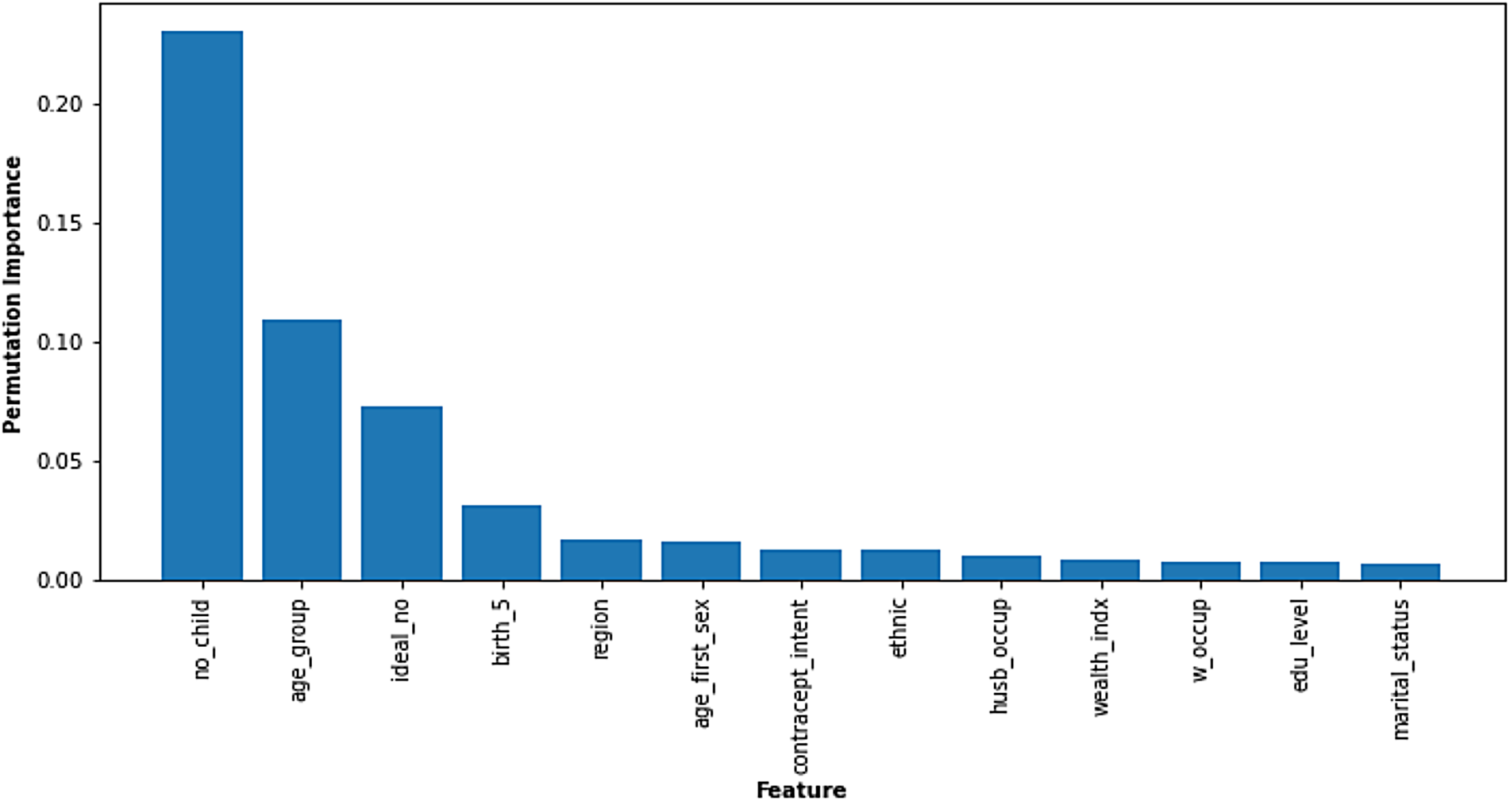
Permutation feature importance. Copyright (c) 2024 Project Jupyter Contributors. All rights reserved. Licensed under BSD-3-Clause.

Permutation feature analysis from the Random Forest model reveals the factors most influential in predicting fertility desire among women in Nigeria (Figure 4). The chart ranks features based on their “permutation importance,” with the *X*-axis representing the feature and the *Y*-axis showing its relative significance. The number of children a woman already has (“no_child”) emerged as the most critical factor, reaffirming its pivotal role in shaping fertility preferences. This was followed closely by a woman's age group (“age_group”) and her ideal number of children (“ideal_no”), which also holds substantial influence over the model's predictions. In addition, features such as the number of births in 5 years (“birth_5”), regional variations (“region”), and age at first sexual intercourse (“age_first_sex”) demonstrate moderate importance. The remaining features, including contraceptive intention, ethnicity, spousal occupation, wealth index, woman's occupation, education level, and marital status, contribute to the model's prediction but to a lesser extent.

#### Gini impurity feature importance

**Figure 5:**
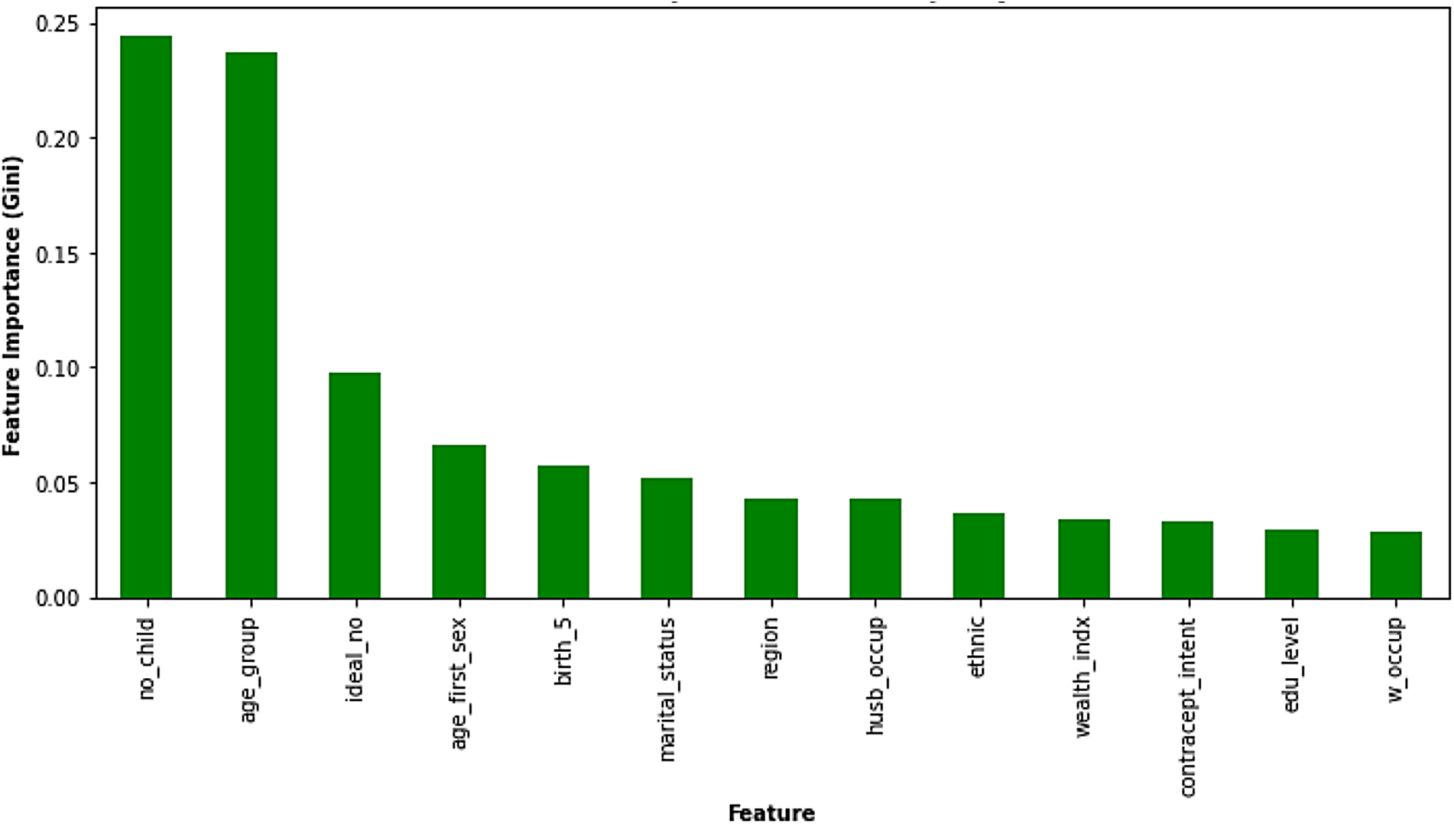
Gini impurity feature importance. Copyright (c) 2024 Project Jupyter Contributors. All rights reserved. Licensed under BSD-3-Clause.

Gini impurity, a measure of node impurity within decision trees, serves as another metric for feature importance in the Random Forest model. The Gini impurity features importance chart (Figure 5) reflects this, depicting a similar pattern to the permutation importance analysis. Features ranked higher on the *X*-axis with greater Gini importance (*Y*-axis) contribute more significantly to the model's predictions. Notably, the number of children a woman already has (“no_child”) emerged as the most influential factor, reaffirming its paramount importance. This was followed closely by a woman's age group (“age_group”) and her ideal family size (“ideal_no”), which also exhibited substantial influence on the model's predictions. In addition, regional variations (“region”), number of births in the last 5 years (“birth_5”), and age at first sexual intercourse (“age_first_sex”) demonstrate moderate importance. While these features prominently shape the model's predictions, others such as contraceptive intention, ethnicity, spousal occupation, wealth index, woman's occupation, education level, and marital status are also considered but with a lesser impact.

## Discussion

This study aimed to predict and identify the predictors of fertility preference among women in Nigeria. A sample of 37,581 women was included from the 2018 NDHS data. This study covers a less-explored area of demographic research methodology by employing machine learning algorithms to predict the fertility preferences of women in Nigeria. Before employing the ML algorithms, a preliminary statistical analysis of the predictors was performed using traditional logistic regression, which served as a starting point. The six machine learning algorithms employed and evaluated for the study include LR, SVM, KNN, DT, RF, and XGBoost. All the models performed well, with accuracy scores ranging from 0.85 for LR to 0.92 for both RF and XGBoost. However, since the best model needed to be chosen, a closer examination revealed trade-offs between each model's strengths and weaknesses. Model selection was guided by the research objectives and, in this study, minimizing false positives, i.e., incorrectly classifying women as preferring to have more children, was paramount due to the implication of misclassification in healthcare and policy interventions (29–32).

Logistic Regression, with a consistency score of 0.85 across all the metrics, provided a foundation. Its critical limitation was its inability to minimize FN (887) which indicates that it might have missed many women who would truly prefer to have more children. This could lead to a misallocation of resources (33, 34). SVM, however, demonstrated a commendable overall performance with accuracy ranging from 0.88 to 0.89, but the model struggled to minimize true positives with FP (629). KNN stood out for its exceptional precision of 0.95 which indicates its strong ability to minimize false positives compared to LR and SVM. This is critical for policy interventions (35). However, it has a lower recall rate of 0.86 which suggests that it might have missed more true positives, exemplifying the trade-offs between precision and recall (29, 32). DT exhibited a balanced performance with scores of around 0.89 for most of the metrics (Figure 2). Notably, it was able to minimize FN (565), offering a good advantage for interpretability (36). However, it is sensitive to data quality and has a tendency of overfitting which requires careful consideration (37). Random Forest and XGBoost emerged as the top performers with the highest accuracy of 0.92 and precision of 0.94. The confusion matrix demonstrated a balanced performance for RF with high TP and fewer misclassifications. It also exhibited a high recall of 0.91 which further emphasized its effectiveness. XGBoost, in comparison, showed a slightly higher recall of 0.92, but RF surpassed it in precision with 0.94 (Figure 2). Similarly, a recent study conducted in Abadan and Khorramshahr (Khuzestan Province, Iran) compared seven ML models to predict the tendency of childbearing and RF was chosen as the best model (38).

While this study primarily focused on the prediction of fertility preferences among Nigerian women using ML algorithms, a comparison with recent state-of-the-art methodologies highlights the robustness of the employed approach. For instance, in a study utilizing ML algorithms SVM, RF, and multi-layer perceptron (MLP) for breast cancer classification, similarly, high accuracy and interpretability were achieved, with MLP attaining an AUC of 99.71% (39). Further comparisons with other research studies have demonstrated the utility of ML techniques in disease prediction and healthcare, emphasizing the value of feature-level insights for improving model interpretability and applicability (40, 41).

The analysis of feature importance with the RF model revealed that the number of living children, woman's age, ideal number of children, number of births in the last 5 years, and regional variations were key predicting factors influencing fertility preferences among Nigerian women. In this study, the number of living children appeared to be the highest predicting factor, which is consistent with previous studies showing that women with a large number of children are more likely to want to limit further childbearing (42, 43). A possible explanation could be that women with a larger number of living children may be satisfied with their current family size or have met their reproductive goals. This finding is also in line with a study from sub-Saharan Africa (44). We observed from this study that a woman's age had a significant contribution to predicting fertility preference. This finding is supported by studies conducted in Ethiopia (45), Iran (46), and Ghana (47) where women aged 15–24 years were more likely to desire more children than other age groups. This could be because younger women have not yet achieved their reproductive goals and are more inclined to want more children later in life (48).

Our study also showed that the ideal number of children is another predicting factor, which aligns with studies conducted in 53 (49) and 78 LMICs from five geographical regions (East and South Africa, Middle and West Africa, Latin America, South and Southeast Asia, and West Asia and North Africa) (50), respectively. Similarly, a study conducted in Ghana (51) indicated that the importance of the ideal number of children resonates with studies that highlight that women who express an ideal number of children tend to strive to achieve their preference (52). Furthermore, other significant factors were educational attainment, marital status, and economic status, which are all known to influence reproductive choices and opportunities, but these had a smaller effect on our findings (51–53). Discrepancies in research scope and setting, the sample population, and the time these studies were conducted are all plausible explanations for the differences in study findings.

## Conclusion

This study explored the application of machine learning algorithms to predict fertility preferences among Nigerian women, offering a novel approach with potential application in family planning interventions. By evaluating and comparing ML models, this study demonstrated the potential of advanced analytical methods in capturing complex interactions between variables that traditional statistical models may ignore. Random Forest emerged as the best model due to its high precision and AUROC values, offering a dependable tool for minimizing false positives in fertility predictions. The analysis of feature importance highlighted key factors such as a woman's current number of children, age group, and ideal family size, which are consistent with existing demographic studies. These findings not only improve our understanding of fertility preferences in Nigeria, but also demonstrate the broader applicability of machine learning techniques in demographic studies, notably for family planning and resource allocation.

### Limitation and strength

This study leverages Demographic and Health Survey (DHS) data which is a valuable resource due to its extensive population coverage. However, DHS data, including the 2018 Nigerian data used for this study, are susceptible to self-reporting biases. Social desirability bias, for example, can influence responses on sensitive topics such as fertility preferences. In addition, we only accounted for the fertility preference of women, but fertility preference could be affected by the preference of a partner. Furthermore, the complexity of DHS data presented challenges during feature engineering, raising the possibility that relevant variables influencing fertility preferences might have been overlooked.

Despite these limitations, the study offers valuable insights. Cross-validation techniques effectively addressed concerns about overfitting, strengthening the confidence we can have in the generalizability of the models. These findings can inform future research and policy interventions aimed at addressing fertility concerns. Furthermore, this study contributes to the existing literature on fertility preference and machine learning.

## Data Availability

The original contributions presented in the study are included in the article/Supplementary Material, further inquiries can be directed to the corresponding author.
